# Adverse Drug Reactions in an Intensive Care Unit of a Secondary Care Lithuanian Hospital: A Prospective Observational Study

**DOI:** 10.3390/healthcare13131592

**Published:** 2025-07-03

**Authors:** Greta Masiliūnienė, Gintautas Gumbrevičius, Edgaras Stankevičius, Edmundas Kaduševičius

**Affiliations:** 1 Institute of Physiology and Pharmacology, Faculty of Medicine, Medical Academy, Lithuanian University of Health Sciences, A. Mickevičiaus 9, LT-44307 Kaunas, Lithuania; gintautas.gumbrevicius@kaunoligonine.lt (G.G.); edgaras.stankevicius@lsmu.lt (E.S.); edmundas.kadusevicius@lsmu.lt (E.K.); 2 Department of Adult Intensive Care and Resuscitation, Kaunas Hospital of the Lithuanian University of Health Sciences, Josvainių 2, LT-47144 Kaunas, Lithuania; 3 Department of Internal Diseases, Kaunas Hospital of the Lithuanian University of Health Sciences, Josvainių 2, LT-47144 Kaunas, Lithuania

**Keywords:** adverse drug reaction, preventability, causality, severity, intensive care unit

## Abstract

**Background and Objectives**: Previous studies have shown that a major part of adverse drug reactions (ADRs) are preventable, and they contribute to increased morbidity, mortality, and costs. To our knowledge, no study investigating preventable ADRs has been carried out in Lithuania. Therefore, the aim of this study was to characterize ADRs in the intensive care unit (ICU) of a secondary care Lithuanian hospital as well as to identify drug classes and organ systems most commonly implicated in preventable and nonpreventable ADRs. **Materials and Methods**: This observational prospective study was conducted in an 18-bed ICU of Kaunas Hospital of the Lithuanian University of Health Sciences from 1 September 2021 to 31 August 2023. All ADRs were assessed for causality, severity, and preventability. The Anatomical Therapeutic and Chemical (ATC) system was used to classify drug classes implicated in ADRs. The organ systems affected were analyzed using the Medical Dictionary for Regulatory Activities (MedDRA). **Results**: A total of 154 patients with a median age of 78.8 years (range, 18–97) were enrolled into this study. There were 255 ADRs identified; preventable ADRs accounted for 87.5%. Among the preventable ADRs, the top three therapeutic subgroups were antithrombotic agents (26.5%), anti-inflammatory and antirheumatic products (22.0%), and blood substitutes and perfusion solutions (20.2%). Meanwhile, among nonpreventable ADRs, antibacterials for systemic use (62.5%) and antithrombotic agents (46.9%) were the two most common therapeutic subgroups. The gastrointestinal as well as the skin and subcutaneous tissues organ systems were more likely to be affected by nonpreventable ADRs (56.3% vs. 17.5%, *p* ˂ 0.05 and 12.5% vs. 0.4%, *p* ˂ 0.05, respectively), while the renal and urinary organ systems were more likely to be affected by preventable ADRs (38.1% vs. 6.3%, *p* ˂ 0.05). **Conclusions**: Our study showed a very high incidence of preventable ADRs (87.5%). Drugs affecting blood and blood-forming organs were most frequently implicated in these ADRs. This area deserves special attention and strategies need to be implemented to reduce the incidence of preventable ADRs and their impact on the healthcare system. Moreover, it emphasizes the need for future studies at a national level as, to our knowledge, this is the first study addressing the issues of avoidable harm at the ICU of one Lithuanian hospital.

## 1. Introduction

Numerous studies, systematic reviews, and meta-analyses carried out during the last three decades have emphasized drug-related issues as being a huge burden on human health and healthcare systems, leading to considerable morbidity, mortality, and costs [1,2,3,4,5]. The use of definitions for these drug-related issues, such as adverse drug reactions (ADRs), adverse drug events (ADEs), and medication errors in studies, is very inconsistent, often depending on researchers’ preference, and makes the comparison of these studies difficult [6,7]. The 2010 European Union (EU) legislation on pharmacovigilance, which came into force in 2012, amended the previous definition of an ADR by the World Health Organization (WHO) [8] and introduced its extended version “to ensure that it covers noxious and unintended effects resulting not only from the authorised use of a medicinal product at normal doses, but also from medication errors and uses outside the terms of the marketing authorisation, including the misuse and abuse of the medicinal product” [9].

ADRs are an important cause of hospital admissions [10] and are among the top causes of death in many countries across the world [11]. The most recent data by the American Society of Pharmacovigilance show that ADEs have become the third leading cause of death in the United States [12], while in the EU, ADRs are recognized as the fifth most common cause of death [13].

Previous systematic reviews and meta-analyses have provided estimates that ADRs are responsible for 5% to 11.2% of all hospital admissions [4,14]. As much as 24% to 88% of ADR-related hospital admissions are considered preventable [15,16,17,18]. Serious ADRs frequently lead to admissions to intensive care units (ICUs), posing an additional burden on healthcare by overloading ICUs and limiting the accessibility of ICUs to critically ill patients. Based on one systematic review that analyzed 11 prospective and retrospective studies, the incidence of ADE-related ICU admissions ranged from 0.37% to 27.4%, and preventable ADEs made up 17.5% to 85.7% [19]. This great variation in the preventable ADR-related hospital and ICU admissions could be explained by differences in the study design, setting, and population, definitions of drug-related events, and tools used to assess preventability [16,17,20].

Patient safety is the primary focus of every healthcare institution, and reducing avoidable harm is one way to improve patient safety [21]. Therefore, it is important to distinguish between preventable and nonpreventable ADRs to allocate available resources to achieve the greatest benefit in reducing the incidence of preventable ADRs [22]. Although this topic has been extensively explored in other countries, as shown in a 2020 meta-analysis [22], to our knowledge, no study specifically addressing the issues of ADRs in Lithuanian hospitals has been published to date. Therefore, the aim of our study was to determine the incidence, causality, and preventability of ADRs in the ICU of a secondary care Lithuanian hospital. Moreover, we aimed to analyze what organ systems were most commonly affected by ADRs, what drug classes and individual drugs were most commonly associated with preventable and nonpreventable ADRs, and if there were any differences in demographic and clinical characteristics, hospital length of stay, and outcomes and costs between patients who experienced preventable and nonpreventable ADRs.

## 2. Materials and Methods

### 2.1. Study Design, Setting, and Population

This prospective observational study was conducted in an 18-bed ICU of Kaunas Hospital of the Lithuanian University of Health Sciences (Kaunas, Lithuania). It is a secondary care multiprofile hospital providing healthcare in surgery, geriatrics, infectious diseases, cardiology, internal medicine, neurology, gynecology and obstetrics, and urology, but not traumas. The study took place between 1 September 2021 and 31 August 2023. All patients newly admitted to the ICU during this period were screened for possible ADRs by employing the Naranjo algorithm [23]. Additionally, all ICU patients were followed prospectively for ADRs that could occur during ICU stay. Two researchers, namely an ICU physician (G.M.) and a clinical pharmacologist (G.G.), were involved in screening and assessing all suspected ADR cases. In case of disagreements, the consensus was reached by discussions.

The study was approved by Kaunas Regional Biomedical Research Ethics Committee (approval no. BE-2-60, dated 18 August 2021). All the patients or their legal representative signed the informed consent form before enrollment into the study.

The following data of patients were collected and entered into a database: sex, age, reasons for ICU admission, prescription drugs used, creatinine clearance, underlying diseases, number of underlying diseases, duration from hospital to ICU admission, length of ICU stay, overall length of stay, and outcome at discharge from the ICU.

The Simplified Acute Physiology Score II (SAPS II) was used to estimate the risk of death in ICU patients within 24 h after admission [24]. The direct costs of each patient’s hospitalization were extracted from the hospital database. For analysis, the mean cost per patient in euros was calculated.

For analysis, patients were stratified into two groups—preventable and nonpreventable—and their characteristics and clinical data were compared. If a patient had several ADRs and if at least one of them was nonpreventable, he/she was assigned to the nonpreventable group for analysis.

### 2.2. Assessment of Adverse Drug Reactions

*Preventability.* Preventability was assessed by the modified Schumock and Thornton criteria [25,26]. All ADRs based on these criteria were classified into three categories, namely definitely preventable, probably preventable, and nonpreventable. At the beginning for ADR analysis, the distribution of ADRs by 3 categories was analyzed. For further analysis, definitely and probably preventable ADRs were combined into one category, namely “preventable”.

*Causality.* The Naranjo algorithm [23] and the World Health Organization-Uppsala Monitoring Center (WHO-UMC) scale [27] were employed to determine the causality of ADRs. The Naranjo algorithm for causality assessment consists of 10 questions with “yes”, “no”, and “do not know” as possible answers. A different score, i.e., −1, 0, +1, or +2, can be assigned to each question. The total score ranges from −4 to +13, and interpretation of the total score is carried out as follows: an ADR is considered as definite if the score is 9 or higher; it considered probable if the score is 5 to 8; it is considered possible if the score is 1 to 4; and it is considered doubtful, if the score is 0 or less [23]. The WHO-UMC system, a globally accepted method for causality assessment, considers clinical–pharmacological aspects of a case history and classifies ADRs into the following 6 categories: certain, probable/likely, possible, unlikely, conditional/unclassified, and unassessable/unclassifiable [27].

*Severity.* The level of ADR severity was assessed by the modified Hartwig scale [25,28]. ADRs were classified as mild (level 1 or 2), moderate (level 3 or 4), or severe (level 5, 6, or 7) [25].

The Anatomical Therapeutic and Chemical (ATC) classification system was employed to code the classes of drugs being involved in an ADR [29]. This system classifies active substances by using a hierarchy with 5 different levels. In our analysis, ADR-related drugs were classified by the 1st level (anatomical or pharmacological group) and the 2nd level (therapeutic subgroup) [29].

ADRs were examined by the organ system affected according to the 27 system organ classes (SOCs) using the codes of the Medical Dictionary for Regulatory Activities (MedDRA) [30]. These 27 SOCs represent parallel axes, a characteristic known as “multiaxiality” that allows a term represented in more than one SOC to be grouped by different classifications, such as etiology or manifestation site [31]. In our study, ADRs were grouped by a manifestation site.

### 2.3. Statistical Analysis

Statistical analysis was performed with the SPSS statistical package (IBM SPSS Statistics, version 30, Chicago, IL, USA) and Excel (Microsoft^®^ Excel^®^ for Microsoft 365 MSO, Version 2503, Redmond, WA, USA). The distribution of continuous data was checked with the Shapiro–Wilk test (in cases where the sample size was less than 50) or the Kolmogorov–Smirnov test. If data were found to be nonnormally distributed, they were expressed as medians with ranges and the groups of nonnormally distributed continuous data were compared with the Mann–Whitney test. If continuous data were normally distributed, means with standard deviations were reported, and they were compared with the two-tailed Student’s *t* test. Categorical data were expressed as numbers with percentages. The chi-square test was employed to compare categorical data. The level of significance was set at *p* ˂ 0.05.

## 3. Results

### 3.1. Characteristics of the Study Population

During the study period, a total of 1778 patients were admitted to the ICU. Of them, 154 (8.7%) patients with a median age of 78.8 years (range, 18–97) who experienced at least 1 ADR were enrolled into this study. There were 73 men (47.4%) and 81 women (52.6%). The median age for men and women was 76.3 years (range, 18–96) and 80.7 years (45–97), respectively. The characteristics of the study population are shown in Table 1.

### 3.2. Preventability, Causality, and Severity, Assessment of All Adverse Drug Reactions

During the study, there were 255 ADRs identified, with 45.5% of the patients having more than one ADR (range 1–4). Definitely preventable ADRs accounted for the greatest percentage that significantly differed from the percentages of probably preventable and nonpreventable ADRs (n = 174, 68.2% vs. n = 49, 19.2% and n = 32, 12.5%, respectively; χ^2^ = 141.48, *p* ˂ 0.001). The causality assessment by the Naranjo scale showed that the majority of the ADRs were rated as probable (n = 183, 71.8%) followed by definite (n = 49, 19.2%) and possible (n = 23, 9%) (χ^2^ = 173.46, *p* ˂ 0.001). Certain, probable/likely, and possible ADRs on the WHO-UMC scale accounted for 64.3% (n = 164), 28.6% (n = 73), and 7.1% (n = 18), respectively (χ^2^ = 127.93, *p* ˂ 0.001). The ADRs of moderate severity made up 63.9% of the total (n = 163), as compared to severe ADRs accounting for 36.1% (n = 92) (χ^2^ = 19.77, *p* ˂ 0.001).

### 3.3. Assessment of ATC Drug Classes and MedDRA-Classified Organ Systems Implicated in Adverse Drug Reactions by Preventability

A total of 68 drugs belonging to 22 ATC drug classes by the second ATC level were related to 255 ADRs. Furthermore, we analyzed the distribution of the top 10 therapeutical groups (second ATC level) and the individual drugs most frequently related to ADRs by preventability (Table 2). For this purpose, definitely preventable and probably preventable ADRs were combined into one group (preventable ADRs, n = 223, 87.5%). The top three therapeutic subgroups among the preventable ADRs were antithrombotic agents (26.5%), anti-inflammatory and antirheumatic products (22.0%), and blood substitutes and perfusion solutions (20.2%), while among nonpreventable ADRs, antibacterials for systemic use and antithrombotic agents were the two most common therapeutic groups (62.5% and 46.9%, respectively).

Table 3 shows associations between MedDRA-classified organ systems and the preventability of ADRs. Nonpreventable ADRs were more frequently associated with gastrointestinal disorders (56.3% vs. 17.5%, *p* ˂ 0.05) and skin and subcutaneous tissues disorders (12.5% vs. 0.4%, *p* ˂ 0.05) than preventable ADRs, while renal and urinary disorders were more likely to occur due to preventable ADRs (38.1% vs. 6.3%, *p* ˂ 0.05).

### 3.4. Comparison of Patients with Preventable and Nonpreventable ADRs

Table 4 shows the comparison of patients with preventable and nonpreventable ADRs. The groups were matched for age and sex. The median number of ADRs per patient was significantly greater in the nonpreventable than the preventable ADR group (2, range 1–4, vs. 1, range 1–4; *p* = 0.006). Moreover, the percentages of patients who experienced 3 or 4 ADRs were significantly greater in the nonpreventable than in the preventable ADR group. Neurological diseases were a significantly more common cause for admission among the patients in the nonpreventable ADR group (21.4% and 6.3%, *p* ˂ 0.05). The overall median length of hospital stay was significantly greater in the nonpreventable than preventable group (18.5 vs. 11 days, *p* = 0.014).

The median cost per patient in euros and the median length of ICU stay were greater in the nonpreventable group than in the preventable group, but the differences were not statistically different (EUR 5296 vs. EUR 2586, *p* = 0.75; and 6 vs. 3 days, *p* = 0.251, respectively).

## 4. Discussion

This is the first study in the ICU of a Lithuanian secondary care hospital focusing on ADRs and their preventability. Our study showed that 8.7% of patients experienced at least one ADR during a 2-year period.

Comparison of the incidences of preventable drug-related events across different studies is complicated due to the high variability in the methods used to assess preventability [32]. In our study, where the Naranjo algorithm was used to assess preventability, 87.5% of the 255 ADRs were found to be preventable. This rate of preventable events is one of the greatest among those reported in other studies and is in agreement with some other studies. In a study by Rivkin, conducted in a medical 12-bed ICU, most ADRs (86%) were also judged as preventable [33]. Another study carried out in the general ICUs of five UK hospitals found that 77% of the iatrogenic events were preventable [34]. Hammerman and Kapeliovich reported that 64% of the iatrogenic events in the cardiac ICU were considered as preventable [35]. Preventable ADEs made up 59% of ICU admissions in a study by Jolivot et al. [36]. However, other studies reported much lower incidences of preventable drug-related events. A study in the medical ICU by Grenouillet-Delacre et al. found that in 48% of cases, ADRs were preventable [37]; Lehmann et al. assessed 34% of the cases to be preventable [38]. Some studies reported very low incidences of drug-related preventable events. A retrospective study by Park et al. in two ICU wards of a tertiary care teaching hospital reported 20% of ADRs being preventable [39]. Nazer et al., in their study involving the medical/surgical ICU of a teaching cancer center, found only 17.5% preventable ADRs [40]. It is worth noting that while analyzing the incidences of preventable events across the studies, we noticed a tendency toward lower incidences being reported in tertiary care hospitals where more severe and complex cases are treated in specialized, such as oncology or hematology, units. The studies mentioned above that were conducted in tertiary care (teaching) hospitals reported lower incidences of preventable drug-related events ranging from 17.5% to 64% [35,36,37,38,39,40], except for the study by Rivkin that indicated a similar percentage of preventable ADRs [33].

In a systematic review by Howard et al. conducted in 2006, the pooled data from nine studies showed that antiplatelets (16%), diuretics (16%), nonsteroidal anti-inflammatory drugs (11%), and anticoagulants (8%) were the drug groups most commonly linked to preventable drug-related hospital admissions [41]. Meanwhile in the study by Grenouillet-Delacre et al., psychotropic drugs (22.5%), immunosuppressive drugs (21.6%), and anticoagulant drugs (13.5%) were the top three drug classes most frequently involved in ICU admission-related ADRs [37]. In a Taiwanese study by Chen et al., carried out in the emergency department of a tertiary referral center, cardiovascular agents were associated with the largest portion of preventable ADEs (32.2%) [42]. This in line with the results of the study by Jolivot et al., where cardiovascular drugs were also the most common medicines implicated in preventable ADEs (30%) [36]. In our study, cardiovascular system drugs ranked second with the percentage being similar (31.2%). The most common drug class associated with preventable ADRs in our study were drugs acting on blood and blood-forming organs (47.1% of all preventable ADRs), where antithrombotic agents (B01) and blood substitutes and perfusion solutions (B05) accounted for 26.5% and 20.2%, respectively. Jolivot’s et al. study indicated drugs acting on blood and blood-forming organs (17%) as the third most common to be involved in preventable ADEs, but the authors did not specify how these drugs were distributed by the second ATC level [36]. To our knowledge, a study by Trunet et al., one of the earliest studies on drug-related reactions in an ICU, is the only one study reporting intravenous solutions as the third most common ADR. They accounted for 12.4% of all drug classes implicated in drug-induced illnesses and were preceded by psychotropic drugs (17.5%) and anticoagulants (13.4%) [43]. However, the authors did not provide any information on whether these intravenous solutions were implicated in preventable or nonpreventable ADRs. In our study, such a great percentage of 0.9% sodium chloride (19.7%) being related to preventable ADRs points to its improper administration. The need for the infusion of larger volumes of isotonic solution per day may increase sodium above physiological levels and lead to hypernatremia [44]. Monitoring critically ill patients who are given larger volumes of saline is necessary due to electrolyte imbalance [44]. Nowadays, balanced crystalloid solutions, where the content of sodium, potassium, and chloride is closer to that of extracellular fluid, are being used as an alternative to saline [45].

Regarding nonpreventable ADRs, information on drugs that caused nonpreventable ADRs is scarce. In the study by Jolivot et al., antineoplastic and immunomodulating agents accounted for 41% of nonpreventable drug-related ADEs, followed by drugs acting on blood and blood-forming organs (23%) and the nervous system (13%) [36]. The analysis of our study showed different drug classes being associated with nonpreventable ADRs as compared with study. Antibacterials for systemic use and antithrombotic agents were implicated in the overwhelming majority of nonpreventable ADRs (62.5% and 46.9%, respectively). This difference in the most common ADR-related drugs could be explained by the fact that in the abovementioned study, the hospital had oncology, hematology, and hepatology wards that could be significant providers of patients with nonpreventable ADEs [36]. However, our findings are in line with the results of the study by Bates et al., who also found antibiotics (30%) along with analgesics (30%) as major contributors to the occurrence of nonpreventable ADEs [46].

When all ADRs were evaluated by organ systems affected, in our study, renal and urinary (34.1%), gastrointestinal (22.4%), and nervous (18.0%) disorders were most common. The same organ systems affected can also be found to be reported among the top three systems by other studies. Jolivot et al. reported renal and urinary disorders (17%), nervous systems disorders (15%), and infections and infestations (14%) as the most common [36]. Meanwhile, central nervous system complications (18%), gastrointestinal complications (18%), allergic/cutaneous complications (16%), and cardiovascular complications (16%) were most frequent in the study by Bates et al. [46]. An Australian study by Li et al. evaluated ADR-related hospital admissions and reported that the top three reported types of ADRs by MedDRA were gastrointestinal system (16%), injury, poisoning, and procedural complications (16%), and renal and urinary system (13.2%) [47].

Numerous studies have confirmed that ADRs are associated with a longer length of hospital stay and increased hospital costs [1,3,48,49,50]. Moreover, in ICU settings, the economic impact of ADRs on costs and the length of stay is more significant. Cullen et al. in their study reported that the length of stay after an ADE in ICU settings was 1.6 days longer and that total costs after an ADE were USD 5691 greater than in non-ICU settings [51]. However, data on the costs of ADRs and length of stay regarding preventable and nonpreventable ADRs in the ICU setting are very limited. In the study by Jolivot et al., the median length of ICU stay was similar between the preventable and unpreventable ADE groups (4 days, IQR 2–7, vs. 4 days, IQR 2–9), but the median length of hospital stay differed significantly between these groups (13 days, IQR 5–28, vs. 23 days, IQR, 9–48) [36]. The findings in our study are in agreement with those mentioned above: although the median length of ICU stay was longer for patients with nonpreventable ADRs than those with preventable ADRs, the difference was not statistically significant (6 days, range 0–42, vs. 3 days, range 0–92). Patients with nonpreventable ADRs in our study stayed significantly longer than those with preventable ADRs (median 18.5 days, range 2–70, vs. 11 days, range 0–142). An analysis of costs in our study revealed no significant difference in the cost per patient between the nonpreventable and preventable groups despite it being greater in the nonpreventable group (median 2586 euros, range 164–25,344 vs. 5296, range 430–13,111). The study by Jolivot et al. showed a similar tendency with no significant difference (median 5802 euros, IQR 2425–13,460 vs. 3688, IQR 2562–8705).

Given that the overwhelming majority of identified ADRs in our study were preventable, different strategies and interventions could be suggested for the hospital healthcare system to reduce the incidence of preventable ADRs. It is well known that different interventions, such as the engagement of a pharmacist as a full member of the patient care team in the ICU [52], the installation and usage of electronic prescribing systems with medication decision support [53], and the implementation of robust ADR and pharmacovigilance education programs for hospital staff [54], lead to a lower rate of preventable ADRs. It is worth noting that at the time when this study was carried out, there was no clinical pharmacologist employed by the hospital, and this may have contributed to a high incidence of preventable ADRs.

Several limitations of our study have to be acknowledged. First, our study was conducted in the ICU of a single secondary care hospital, and based on the findings of our study, no generalization can be made regarding the incidence of ADRs and their characteristics in the ICU settings of other hospitals. A high degree of variation across hospitals treating different patient populations and specializing in various areas is possible. Second, in our study, we did not categorize ADRs by the classification initially proposed by Rawlins and Thompson in 1977. Third, selection bias might exist, as two investigators screened all possible ADR cases. Fourth, the study lacks the analysis of DDIs, and this could be a direction for future research. Finally, this study was initiated during the COVID-19 pandemic, and its contribution to the complexity of cases remains unclear.

## 5. Conclusions

The results of our study conducted at the ICU of the secondary care hospital showed a very high incidence of preventable ADRs (87.5%). Drugs affecting blood and blood-forming organs were most frequently implicated in these ADRs. This deserves special attention and strategies need to be implemented to reduce the incidence of preventable ADRs and their impact on the healthcare system. Moreover, it emphasizes the need for future studies at a national level as, to our knowledge, this is the first study addressing the issues of avoidable harm in the ICU of one Lithuanian hospital.

## Figures and Tables

**Table 1:** Characteristics of the study population (n = 154).

Characteristic	Values
Sex, n (%) Men Women	73 (47.4) 81 (52.6)
Age, years, median (range)	78.81 (18–97)
Age groups, n (%) 18–44 years 45–64 years >65 years	6 (3.9) 18 (11.7) 130 (84.4)
Number of ADRs per patient, median (range)	1 (1–4)
Number of ADRs, n (%) 1 2 3 4	84 (54.5) 44 (28.6) 20 (13.0) 6 (3.9)
Number of underlying diseases, median (range) *	2 (0–5)
Number of underlying diseases, n (%) * 0 1 2 3 >3	7 (4.5) 32 (20.8) 44 (28.6) 42 (27.3) 26 (16.9)
Underlying diseases, n (%) * Cardiovascular risk factors Chronic heart failure Chronic renal failure Chronic respiratory disease Neurological disease Psychiatric disease	134 (80.7) 79 (51.3) 35 (22.7) 38 (24.7) 55 (35.7) 20 (13.0)
Creatinine clearance, n (%) >50 mL/min 30–50 mL/min 10–29 mL/min <10 mL/min or hemodialysis	56 (36.4) 30 (19.5) 39 (25.3) 24 (18.8)
Main causes for ICU admission, n (%) Cardiological Acute respiratory insufficiency Metabolic Neurological Shock	26 (16.9) 46 (29.9) 15 (9.7) 14 (9.1) 53 (34.4)
SAPS II score, mean (SD)	35.47 (12.4)
SAPS II score, n (%) ˂30 30–65 >65	53 (34.4) 99 (64.3) 2 (1.3)
Death, n (%) No Yes	92 (59.7) 62 (40.3)
Duration from hospital admission to ICU admission, days, median (range)	0 (0–43)
Length of ICU stay, days, median (range)	3 (0–92)
Overall length of stay, days, median (range)	12 (0 –142)
Cost per patient, euros, median (range)	2918 (164–25,344)
Number of medicines used, n (%) * 0 1 2–3 4–5 >5 Missing	2 (1.3) 4 (2.6) 15 (9.7) 21 (13.6) 109 (70.8) 3 (1.9)
COVID-19 Yes No	46 (29.9) 108 (70.1)

* There were three missing cases. ADR, adverse drug reaction; ICU, intensive care unit, SAPS II, Simplified Acute Physiology Score II.

**Table 2:** Top 10 drug classes by the second ATC level and individual drugs most commonly related to ADRs by preventability *.

Drug Class by ATC and Individual Drug	Preventable ADRs, n (%) (n = 223)	Nonpreventable ADRs, n (%) (n = 32)
Antithrombotic agents (B01) Nadroparin Warfarin Aspirin Rivaroxaban	59 (26.5) 23 (10.3) 13 (5.8) 7 (3.1) 4 (1.8)	15 (46.9) 10 (31.3) 0 (0.0) 0 (0.0) 5 (15.6)
Blood substitutes and perfusion solutions (B05) 0.9% sodium chloride Poly(O-2-hydroxyethyl) starch	45 (20.2) 44 (19.7) 1 (0.4)	0 (0.0) 0 (0.0) 0 (0.0)
Cardiac therapy (C01) Amiodarone Digoxin Propafenone	15 (6.7) 8 (3.6) 6 (2.7) 1 (0.4)	0 (0.0) 0 (0.0) 0 (0.0) 0 (0.0)
Diuretics (C03) Spironolactone Torasemide	16 (7.2) 9 (4.0) 7 (3.1)	0 (0.0) 0 (0.0) 0 (0.0)
Beta-blocking agents (C07) Metoprolol Bisoprolol	14 (6.3) 10 (4.5) 4 (1.8)	0 (0.0) 0 (0.0) 0 (0.0)
Agents acting on the renin–angiotensin system (C09) Captopril Zofenopril	7 (3.1) 3 (1.3) 2 (0.9)	0 (0.0) 0 (0.0) 0 (0.0)
Antibacterials for systemic use (J01) Vancomycin Cefuroxime Ceftriaxone	34 (15.2) 10 (4.5) 8 (3.6) 4 (1.8)	20 (62.5) 0 (0.0) 9 (28.1) 2 (6.3)
Anti-inflammatory and antirheumatic products (M01) Ibuprofen Ketoprofen Diclofenac	49 (22.0) 22 (9.9) 18 (8.1) 7 (3.1)	0 (0.0) 0 (0.0) 0 (0.0) 0 (0.0)
Psycholeptics (N05) Alprazolam Quetiapine Bromazepam	28 (12.6) 7 (3.1) 6 (2.7) 5 (2.2)	0 (0.0) 0 (0.0) 0 (0.0) 0 (0.0)
Psychoanaleptics (N06) Mirtazapine Vinpocetine	13 (5.8) 5 (2.2) 4 (1.8)	0 (0.0) 0 (0.0) 0 (0.0)

* Percentages of top 10 drug classes and most frequently implicated ADR-related drugs were calculated from the respective numbers of preventable (n = 223) and nonpreventable (n = 32) ADRs.

**Table 3:** MedDRA-classified organ systems and reactions associated with preventable and nonpreventable ADRs.

MedDRA-Classified Organ System Affected by ADRs	No. (%) of Preventable ADRs (n = 223)	No. (%) of Nonpreventable ADRs (n = 32)	*p* Value
Blood and lymphatic system disorders	6 (2.7)	0 (0.0)	>0.05
Coagulation factors disorder	6 (2.7)	0 (0.0)	
Cardiac disorders	22 (9.9)	0 (0.0)	>0.05
Bradycardia	20 (7.8)	0 (0.0)	
Asystole	1 (0.4)	0 (0.0)	
Prolongation of QTC	1 (0.4)	0 (0.0)	
Endocrine disorders	2 (0.9)	0 (0.0)	>0.05
Hypoglycemia	2 (0.9)	0 (0.0)	
Gastrointestinal disorders	39 (17.5)	18 (56.3)	˂0.05
Gastrointestinal bleeding	27 (12.1)	7 (21.9)	
Diarrhea	12 (5.4)	11 (34.4)	
Hepatobiliary disorders	2 (0.9)	0 (0.0)	>0.05
Liver failure	2 (0.9)	0 (0.0)	
Nervous system disorders	42 (18.8)	4 (12.5)	>0.05
Disorientation	34 (13.3)	0 (0.0)	
Intracranial bleeding	6 (2.7)	3 (9.4)	
Coma	2 (0.8)	0 (0.0)	
Agitation	0 (0.0)	1 (0.4)	
Renal and urinary disorders	85 (38.1)	2 (6.3)	˂0.05
Renal insufficiency	41 (18.4)	2 (6.3)	
Homeostasis disorder	40 (17.9)	0 (0.0)	
Urinary tract bleeding	4 (1.8)	0 (0.0)	
Respiratory, thoracic and mediastinal disorders	18 (8.1)	3 (9.4)	>0.05
Respiratory tract bleeding	13 (5.8)	3 (9.4)	
Pulmonary oedema	4 (1.8)	0 (0.0)	
Respiratory function insufficiency	1 (0.4)	0 (0.0)	
Skin and subcutaneous tissue disorders	1 (0.4)	4 (12.5)	˂0.05
Subcutaneous hematoma	1 (0.4)	1 (3.1)	
Rash	0 (0.0)	3 (9.4)	
Vascular disorders	6 (2.7)	1 (3.1)	>0.05
Extravasation	5 (2.2)	1 (3.1)	
Adrenal cortex insufficiency	1 (0.4)	0 (0.0)	

Values are number (percentage). χ^2^ = 42.45; df = 9; *p* ˂ 0.001.

**Table 4:** Characteristics of patients by preventability groups.

Characteristic	Preventable ADR Group (n = 126)	Nonpreventable ADR Group (n = 28)	*p*
Sex, n (%) Men Women	62 (49.2) 64 (50.8)	11 (39.3) 17 (60.7)	0.342
Age, years, median (range)	79.53 (18–96)	75.55 (27–97)	0.143
Age groups, n (%) 18–44 years 45–64 years >65 years	4 (3.2) 13 (10.3) 109 (86.5)	2 (7.1) 5 (17.9) 21 (75.0)	0.301
Number of ADRs per patient, median (range)	1 (1–4)	2 (1–4)	0.006
Number of ADRs, n (%) 1 2 3 4	74 (58.7) 36 (28.6) 13 (10.3) 3 (2.4)	10 (35.7) 8 (28.6) 7 (25.0) 3 (10.7)	0.018 ˂0.05 >0.05 ˂0.05 ˂0.05
Number of underlying diseases, median (range) *	3 (0–6)	2 (0–6)	0.189
Number of underlying diseases, n (%) * 0 1 2 3 >3	5 (4.1) 25 (20.3) 35 (28.5) 37 (30.1) 21 (17.1)	2 (7.1) 7 (25.0) 9 (32.1) 5 (17.9) 5 (17.9)	0.728
Creatinine clearance, n (%) >50 mL/min 30–50 mL/min 10–29 mL/min <10 mL/min or HD	44 (34.9) 28(22.2) 30 (23.8) 24 (19.0)	12 (42.9) 2 (7.1) 9 (32.1) 5 (17.9)	0.294
Main causes for ICU admission, n (%) Cardiological Acute respiratory insufficiency Metabolic Neurological Shock	26 (20.6) 36 (28.6) 12 (9.5) 8 (6.3) 44 (34.9)	0 (0.0) 10 (35.7) 3 (10.7) 6 (21.4) 9 (32.1)	0.017 ˂0.05 >0.05 >0.05 ˂0.05 >0.05
SAPS II score, mean (SD)	36.17 (12.70)	32.54 (9.18)	0.154
SAPS II score, n (%) ˂30 30–65 >65	41 (32.5) 83 (65.9) 2 (1.6)	12 (42.9) 16 (57.1) 0 (0.0)	0.491
Death, n (%) No Yes	74 (58.7) 52 (41.3)	18 (64.3) 10 (35.7)	0.588
Duration from hospital admission to ICU admission, days, median (range)	0 (0–43)	1.5 (0–18)	0.146
Length of ICU stay, days, median (range)	3 (0–92)	6 (0–42)	0.251
Overall length of hospital stay, days, median (range)	11 (0 –142)	18.5 (2–70)	0.014
Cost per patient, euros, median (range)	2586 (164–25,344)	5296 (430–13,111)	0.75
Number of prescription medicines used, n (%) * 0 1 2–3 4–5 >5	0 (0.0) 3 (2.4) 10 (8.1) 19 (15.4) 91 (74.0)	2 (7.1) 1 (3.6) 5 (17.9) 2 (7.1) 18 (64.3)	0.026 ˂0.05 >0.05 >0.05 >0.05 >0.05
COVID-19 Yes No	38 (30.2) 88 (69.8)	8 (28.6) 20 (71.4)	0.87

* There were three missing cases in the preventable group. HD, hemodialysis.

## Data Availability

The data that support the findings of this study are available from the corresponding author upon reasonable request. The data are not publicly available due to ethical restrictions.

## Abbreviations

The following abbreviations are used in this manuscript:

ADR	Adverse drug reaction
ADE	Adverse drug event
EU	European Union
WHO	World Health Organization
ICU	Intensive care unit
SOC	System organ class
MedDRA	Medical Dictionary for Regulatory Activities
SAPS II	Simplified Acute Physiology Score II
UMC	Uppsala Monitoring Centre
